# Federated Security for Privacy Preservation of Healthcare Data in Edge-Cloud Environments

**DOI:** 10.3390/s25165108

**Published:** 2025-08-17

**Authors:** Rasanga Jayaweera, Himanshu Agrawal, Nickson M. Karie

**Affiliations:** School of Electrical Engineering, Computing and Mathematical Sciences, Curtin University, Bentley, WA 6102, Australia; himanshu.agrawal@curtin.edu.au (H.A.); nickson.karie@curtin.edu.au (N.M.K.)

**Keywords:** homomorphic encryption, federated learning, privacy-preserving, healthcare data

## Abstract

Digital transformation in healthcare has introduced data privacy challenges, as hospitals struggle to protect patient information while adopting digital technologies such as AI, IoT, and cloud more rapidly than ever before. The adoption of powerful third-party Machine Learning as a Service (MLaaS) solutions for disease prediction has become a common practice. However, these solutions offer significant privacy risks when sensitive healthcare data are shared externally to a third-party server. This raises compliance concerns under regulations like HIPAA, GDPR, and Australia’s Privacy Act. To address these challenges, this paper explores a decentralized, privacy-preserving approach to train the models among multiple healthcare stakeholders, integrating Federated Learning (FL) with Homomorphic Encryption (HE), ensuring model parameters remain protected throughout the learning process. This paper proposes a novel Homomorphic Encryption-based Adaptive Tuning for Federated Learning (HEAT-FL) framework to select encryption parameters based on model layer sensitivity. The proposed framework leverages the CKKS scheme to encrypt model parameters on the client side before sharing. This enables secure aggregation at the central server without requiring decryption, providing an additional layer of security through model-layer-wise parameter management. The proposed adaptive encryption approach significantly improves runtime efficiency while maintaining a balanced level of security. Compared to the existing frameworks (non-adaptive) using 256-bit security settings, the proposed framework offers a 56.5% reduction in encryption time for 10 clients and 54.6% for four clients per epoch.

## 1. Introduction

The healthcare sector consistently faces the highest vulnerability to data breaches among all industries worldwide, and Australia has experienced particularly severe incidents. Recent statistics show that healthcare leads all sectors in data-breach incidents, as documented in Australia’s latest notifiable data breaches report [1]. The 2023 MediSecure breach, affecting 12.9 million individuals, represents one of the largest security failures in Australian healthcare history, highlighting the need for enhanced privacy-preserving mechanisms in medical data management [2].

**Figure 1:**
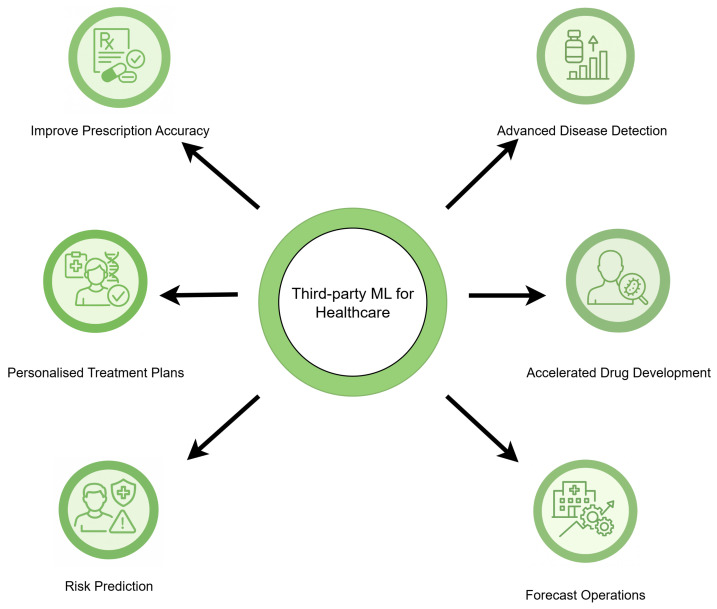
MLaaS use cases in Healthcare Systems.

The digital transformation of healthcare services with the advancement of artificial intelligence has introduced unprecedented challenges to data confidentiality and security, despite being necessary for improvements. Healthcare data must adhere to regulatory frameworks, including GDPR, HIPAA, and equivalent Australian standards like Privacy Act 1988, My Health Record Act [3]. However, using machine-learning applications in healthcare presents a fundamental challenge. While sensitive data can be encrypted for secure storage, most machine-learning algorithms require plaintext data for model training and inference operations. This limitation becomes particularly problematic when institutions use external Machine Learning as a Service (MLaaS), which often have superior computational resources and powerful models but introduce additional security and privacy vulnerabilities. As illustrated in Figure 1. MLaaS provides several advancements to the health sector.

**Figure 2:**
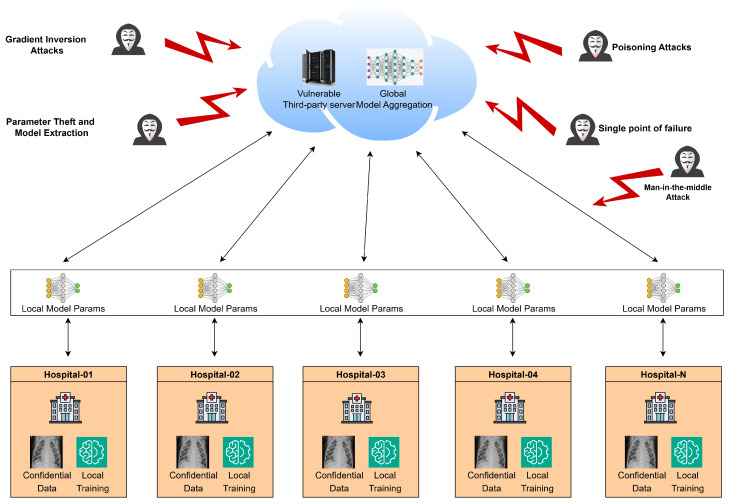
Security vulnerabilities in Federated learning.

Collaborative machine learning presents critical advancements for healthcare institutions. Individual hospitals and medical centers typically maintain limited datasets, which constrains the performance of the model. Decentralized learning approaches that aggregate data from multiple sources enable comprehensive pattern recognition and improved classification accuracy. Such collaboration requires methodologies that preserve data privacy while facilitating effective model training. This capability is achieved by secure collaborative learning using federated learning, where model parameters rather than raw data are shared among participants. However, recent surveys have revealed that gradient updates exchanged during federated learning are vulnerable to reverse engineering attacks [4,5,6]. Among these, gradient leakage attacks and model inversion attacks, as illustrated in Figure 2, allow malicious parties to reconstruct sensitive training data from shared model parameters, compromising the privacy guarantees of federated learning.

Homomorphic encryption emerges as a promising solution to this scenario, allowing mathematical operations to be performed directly on encrypted data without requiring decryption. This includes tensor manipulations that are fundamental to machine-learning algorithms. Earlier research predominantly explored additive homomorphic encryption schemes like Paillier [7,8]. Recent studies have adopted advanced homomorphic encryption schemes such as CKKS and BFV in medical image analysis [9]. However, a comprehensive comparison of these schemes specifically in healthcare datasets remains largely underexplored. Although promising, existing HE-integrated FL systems rely mostly on static encryption schemes with fixed security levels that do not adapt to the computational or communication demands of different model layers. This fixed approach can introduce significant overheads, limiting its real-world applicability in latency-sensitive edge clinical environments.

This paper addresses these limitations by introducing the HEAT-FL framework. HEAT-FL introduces a dynamic encryption strategy that selectively tunes the security level of the model parameters based on the sensitivity of the layers and the number of model parameters, thus optimizing the trade-off between privacy, accuracy, and computational efficiency. HEAT-FL is also designed to defend against adversaries attempting gradient leakage or model inversion attacks by ensuring that all client updates are encrypted with parameter tensor-specific CKKS contexts. In our threat model, we assume that clients are not colluding with each other. However, our design partially mitigates risks from colluding malicious clients by preventing them from accessing plaintext model updates of others, and protects against server-side compromise by enforcing encryption throughout aggregation. Our primary contributions include:

1. Propose and evaluate a Homomorphic Encryption-based Adaptive Tuning for Federated Learning framework (HEAT-FL) for model parameters that outperform traditional fixed security levels while significantly reducing computational overhead through dynamic parameter selection. Compared to the fixed 256-bit security setting, it achieves a 56.5% reduction in encryption time for 10 clients and 54.6% for 4 clients per one federated round.
2. Comprehensive performance analysis using the Chest X-ray Dataset for pneumonia classification and the MRI dataset for brain tumor detection across varying encryption schemes, client configurations, and model architectures.

To maintain compliance with privacy regulations such as GDPR, HIPAA, and Australia’s Privacy Act 1988, the proposed HEAT-FL framework utilizes end-to-end encryption of model parameters using homomorphic encryption. Since raw data remains within client devices and only encrypted model gradients are transmitted, HEAT-FL satisfies the key data minimization, integrity, and confidentiality principles outlined in Article 5 of GDPR [10]. Furthermore, the framework aligns with HIPAA’s requirements for the de-identification of Protected Health Information (PHI) by ensuring that no individually identifiable health data are ever transmitted outside client devices [11].

The remainder of this paper proceeds as follows: Section 2 reviews related work in privacy-preserving healthcare analytics. Section 3 presents the preliminaries for our approach. Section 4 provides details of the experimental methodology. Section 5 evaluates performance metrics in the results section. Section 6 presents the discussion, while Section 7 provides the conclusions and future research directions.

## 2. Related Work

The use of federated learning for privacy preservation has gained significant research attention in recent years [7,12]. This section reviews current approaches in privacy-preserving federated learning, focusing on homomorphic encryption techniques and their application to healthcare data in distributed environments. Conventional machine-learning techniques require data aggregation at a central location to manage multiple datasets, creating vulnerabilities at a single point. Federated learning has emerged as a promising technique for collaborative machine learning without exposing raw data to centralized servers [13]. However, recent studies have shown that traditional federated learning approaches still face substantial privacy risks. Park and Lim identified that attackers can exploit gradient information to access sensitive data, even when the raw data never leaves local devices [12]. Their analysis revealed that centralized servers or malicious participants can potentially extract private information from local model parameters.

Wang et al. developed PPFLHE, a federated learning framework that combines Paillier homomorphic encryption with client-side access control to protect healthcare data while maintaining accuracy [7]. Their approach addresses both external and internal threats by verifying client identities through an access control mechanism and incorporating an Acknowledgment (ACK) protocol to reduce latency and overhead caused by unreliable clients. Testing on medical imaging datasets showed strong classification accuracy (81.53%) with better communication efficiency, proving that homomorphic encryption can work effectively in real-world federated settings. The PPFLHE framework relies solely on Paillier encryption, which supports only additive operations, thereby limiting its ability to perform complex computations.

Fang and Qian proposed PFMLP, a multi-party privacy-preserving machine-learning framework that combines federated learning with partially homomorphic encryption [8]. Their scheme encrypts gradients during training to protect against member inference attacks, achieving near-equivalent accuracy compared to unencrypted training. Notably, they optimized the standard Paillier algorithm to reduce encryption time overhead by approximately 25–28%, addressing one of the critical drawbacks of HE in federated systems. The authors also investigated trade-offs between key length, encryption performance, and security, offering valuable insights for scalable deployment. This framework also relies solely on Paillier encryption, creating gaps such as a lack of support for floating-point data and limited applicability to real-world machine-learning models that require complex arithmetic operations.

In addition, Park and Lim introduced a privacy-preserving federated learning algorithm that enables encrypted model parameter aggregation across devices using a distributed cryptosystem [12]. Unlike other works that rely on a shared key among participants, their approach supports independent key management per client, reducing the risk of key compromise. This architecture ensures that even if one client’s key is exposed, the privacy of the broader FL system remains intact. Their work is particularly relevant to large-scale edge environments, where heterogeneous devices may have varying security capabilities. This work does not compare alternative homomorphic encryption schemes and applies a uniform encryption strategy across all model layers, leading to suboptimal performance in federated healthcare applications.

Lessage et al. [9] implemented a secure federated learning framework using fully homomorphic encryption (FHE) to train deep models on sensitive mammography data without compromising patient privacy. Their system integrates the CKKS scheme between clients and a central server, encrypting the neural network’s last layers during training using a fixed encryption context. Experiments on a breast cancer detection dataset showed that the homomorphically encrypted model achieved nearly the same performance as unencrypted training. The approach is constrained by the substantial memory overhead of FHE, highlighting practical limits on encrypting entire models.

Pan et al. [14] developed FedSHE, a federated learning framework that uses adaptive segmented CKKS homomorphic encryption to secure gradient exchange while preserving model accuracy. Their design reduces computational overhead by selectively encrypting sensitive model layers, showing promising results in benchmark image classifications. Their approach addresses CKKS’s practical limitations by selecting encryption parameters to balance security and efficiency, and introducing an adaptive segmentation technique that splits large model updates into smaller ciphertexts, overcoming CKKS’s encryption length constraint. Experiments on benchmarks like MNIST and CIFAR-10 showed that FedSHE achieves high accuracy (99.2% on MNIST) comparable to plaintext training, with faster computation and lower communication overhead than prior HE-based FL methods, demonstrating the practicality of optimized fully homomorphic encryption in federated settings. Unlike FedSHE, which uses a single CKKS context for the whole model and segments large layers, HEAT-FL adaptively selects CKKS context for each layer based on its sensitivity and size, providing more granular privacy control.

All these studies indicate the wide adoption of privacy-preserving techniques in federated learning. Most existing methods either rely on fixed encryption schemes or address specific aspects of the FL pipeline. The challenge remains in optimizing these cryptographic techniques to reduce computational and communication overhead, particularly within resource-constrained edge–cloud ecosystems.

Our work is motivated by valuable findings and research gaps in the literature. We build on insights from the above studies to further improve the trade-offs between privacy, accuracy, and efficiency. To achieve these advancements, our work focuses on homomorphic encryption schemes like CKKS and BFV, while our adaptive encryption approaches aim to reduce encryption overhead. This related work section provides the context and motivation for our contributions, as we address the open challenge of robust, privacy-preserving federated learning for medical AI at the edge.

## 3. Preliminaries

This section establishes the mathematical basis and principles underlying the privacy-preserving federated learning framework. We formalize the integration of federated learning with homomorphic encryption to enable secure multi-party computation while maintaining model performance.

### 3.1. Homomorphic Encryption Schemes

Homomorphic encryption (HE) enables computation on encrypted data without requiring decryption [15]. Based on the type and number of supported mathematical operations, homomorphic encryption schemes can be categorized into four distinct classes. Partially homomorphic encryption (PHE) enables either additive or multiplicative computations on encrypted data. Somewhat Homomorphic Encryption (SHE) and Leveled Fully Homomorphic Encryption (LHE) permit arbitrary additions and a bounded number of multiplications on ciphertexts. Fully homomorphic encryption (FHE) represents the most advanced form, supporting unrestricted arithmetic operations without computational limits [15]. Modern LHE schemes constructed from the Ring-Learning With Errors (RLWE) assumption can achieve full homomorphic capabilities through bootstrapping techniques [16]. Let E denote an encryption function and D a decryption function. For input values $m_{1}$ and $m_{2}$, a homomorphic encryption scheme satisfies:

(1) $$D\left(E\left(m_{1}\right)⊕E\left(m_{2}\right)\right)=m_{1}+m_{2}$$

where ⊕ represents the homomorphic addition operation on ciphertexts. The mathematical notations and symbols applied in the equations of Section 3.1 are summarized in Table 1. Our framework leverages two prominent HE schemes from the TenSEAL library. A summary of the cryptographic schemes considered across this study is shown in Table 2.

#### 3.1.1. Paillier Homomorphic Encryption

The Paillier cryptosystem is a Partially Homomorphic Encryption (PHE) scheme that supports additive homomorphic operations, exhibiting limitations in federated learning contexts. Unlike CKKS, Paillier only supports integer operations and lacks native support for tensor computations, requiring costly conversions and reduced precision when applied to neural network parameters [18].

#### 3.1.2. CKKS Scheme

The Cheon–Kim–Kim–Song (CKKS) scheme enables approximate arithmetic on encrypted real numbers and supports both addition and multiplication operations [19]. For vector inputs $x=(x_{1},\;x_{2},\;\ldots ,\;x_{n})$ and $y=(y_{1},\;y_{2},\;\ldots ,\;y_{n})$, CKKS satisfies:

(2) D(E(x)⊕E(y))≈x+y

(3) D(E(x)⊗E(y))≈x⊙y

where ⊙ denotes element-wise multiplication. The CKKS scheme employs a rescaling technique to manage the growth of noise during homomorphic operations, defined as:

(4) $$\Delta^{-1}\cdot \left\lfloor \Delta \cdot m\right\rceil +e\approx m$$

where Δ is a scaling factor and *e* represents the error term. This approximation enables efficient operations on floating-point values with controlled precision loss, making CKKS particularly suitable for neural network parameter encryption [19].

#### 3.1.3. BFV Scheme

The Brakerski–Fan–Vercauteren (BFV) scheme provides exact arithmetic on integers [20]. Unlike CKKS, BFV guarantees exact results for homomorphic operations:

(5) $$D\left(E\left(m_{1}\right)⊕E\left(m_{2}\right)\right)=m_{1}+m_{2}$$

(6) $$D\left(E\left(m_{1}\right)⊗E\left(m_{2}\right)\right)=m_{1}\cdot m_{2}$$

BFV relies on the Ring-Learning With Errors (RLWE) problem for security, with ciphertexts defined as:

(7) $$c=\left(c_{0},c_{1}\right)=\left(a\cdot s+m+e\;\mathrm{mod}\;q,-a\;\mathrm{mod}\;q\right)$$

where *s* is the secret key, *a* is uniformly random, *e* is a small error term, and *q* is the ciphertext modulus [21].

### 3.2. Privacy-Preserving Federated Learning

Federated learning enables collaborative model training across multiple clients without sharing raw data. The process is formalized as:

(8) $$F\left(w\right)=\sum_{k=1}^{K}\frac{n_{k}}{n}F_{k}\left(w\right)$$

Here, $F_{k}\left(w\right)$ represents the local objective function for client *k* with $n_{k}$ data points, and $n=\sum_{k=1}^{K}n_{k}$ is the total number of data points across all clients. This represents the weighted average of the local objective function across all clients. Each client’s contribution to the global objective is considered in the learning process [4].

### 3.3. Privacy-Preserving Federated Learning Model Parameter Framework

**Figure 3:**
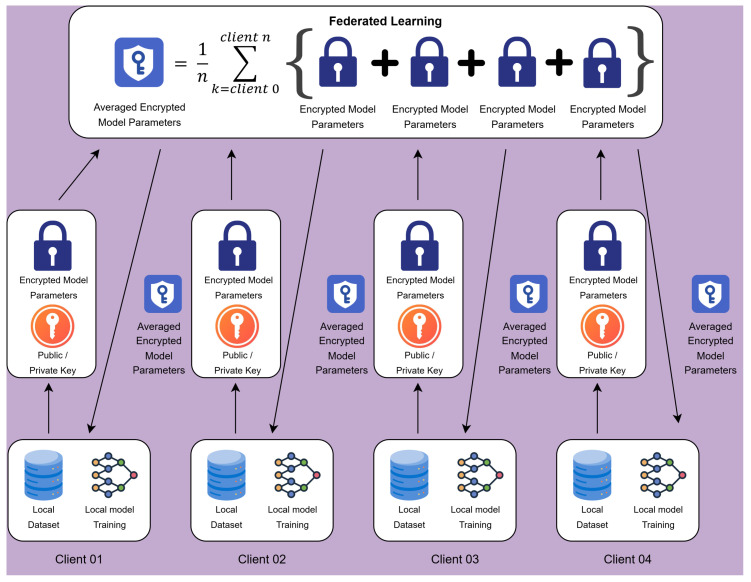
System architecture for a privacy-preserving federated learning model parameter framework.

The Privacy-Preserving Federated Learning Model Parameter framework (PPFLMP) described in Algorithm 1 integrates homomorphic encryption with federated learning as illustrated in Figure 3, ensuring secure model aggregation. The key operations include:
**Algorithm 1:** PPFLMP
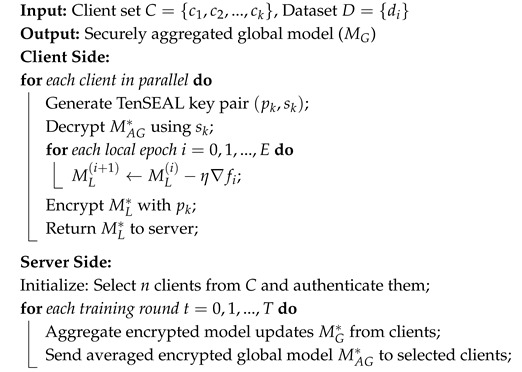


**Local Model Encryption**: Homomorphic encryption enables operations on encrypted data, producing results equivalent to those performed on plaintext. However, traditional homomorphic encryption methods, such as the Paillier homomorphic encryption scheme, present inherent limitations due to their lack of support for division and floating-point or tensor operations, making them inefficient for model aggregation and normalization in federated learning [22]. Paillier only allows additive homomorphism and scalar multiplication on integers, which significantly restricts its usability in federated learning models that rely on floating-point values.

To overcome these challenges, we use the CKKS and BFV scheme in TenSEAL. To achieve secure local model encryption, we configure using officially experimented encryption parameters that balance security and computational efficiency (Table 3). A larger polynomial modulus degree increases security by making the RLWE problem harder, while the coefficient modulus bit sizes must be correspondingly increased to maintain both security balance and sufficient numerical precision [23].

The PPFLMP framework shown ensures that local model updates are encrypted before transmission, preventing data leakage and unauthorized access. The encrypted model updates are aggregated on the central server using secure computation techniques. Initially, data owners train the local models ($M_{L}$) on their dataset (*D*). Once the secure aggregation is finished, each client begins by receiving the encrypted averaged global model parameters ($M_{AG}^{*}$) from the central server. Using the private key ($s_{k}$), the client decrypts the model to obtain the readable parameters. The client then trains its local model ($M_{L}$) on its dataset (*D*) using a computationally efficient model architecture. Once training is completed, the client encrypts the updated model parameters ($M_{L}^{*}$) using the public key ($p_{k}$) and securely uploads them back to the server.

**Aggregation**: Upon receiving encrypted model updates ($M_{L}^{*}$) from multiple clients, the central server performs secure aggregation ($M_{G}^{*}$) without decryption, leveraging homomorphic encryption to ensure privacy preservation. This step prevents the server from accessing individual client models while still being able to compute aggregated global updates.

**Model Update**: The server aggregates the securely updated parameters ($M_{L}^{*}$) from all clients to generate a new averaged encrypted global model ($M_{AG}^{*}$). This updated global model is then distributed back to all clients for the next round of training. The training and aggregation process continues iteratively until a predefined stopping criterion, such as a specific number of training rounds (*T*), is reached, ensuring a robust and privacy-preserving federated learning model.

**Decryption**: In federated learning, decryption is a critical step that ensures secure access to the trained global model while preserving data confidentiality. Each participating client, equipped with the private key ($s_{k}$), receives the encrypted global model parameters ($M_{AG}^{*}$) from the central server. By using the private key, the encrypted values are transformed back into plaintext, allowing the client to retrieve the updated model securely.

## 4. Methodology

### 4.1. Homomorphic Encryption-Based Adaptive Tuning for Federated Learning

Homomorphic Encryption-based Adaptive Tuning for Federated Learning framework (HEAT-FL) applies CKKS homomorphic encryption selectively based on the sensitivity and quantity of model parameters in each layer. The encryption context is dynamically chosen by evaluating the size of each layer. Layers with a small number of model parameters, such as activation function layers, typically representing biases or shallow layers, are encrypted using lightweight context to reduce overhead. Meanwhile, more sensitive layers, such as initial feature extraction layers, receive stronger encryption settings to preserve security. These encryption contexts are configured and managed through shared keys, allowing clients to encrypt their model updates using a suitable context and embed context identifiers within each encrypted layer. This enables the server to perform secure aggregation by matching compatible ciphertexts without decrypting them. Through this adaptive strategy, the system maintains a balance between computational efficiency and privacy preservation, optimizing resource use while ensuring the security of sensitive model information during federated training.

**Local Model Encryption**: Each client performs local training on its private data to obtain an updated local model $M_{L}$. Before sending any information out, the client encrypts its model using the CKKS FHE scheme. A CKKS context is instantiated with appropriate cryptographic parameters to meet a targeted security level and precision requirements. Importantly, HEAT-FL uses adaptive, layer-wise encryption. Each model layer is encrypted under a context optimized for that layer’s characteristics. Formally, for each layer, the client selects encryption parameters that are “context-aware,” meaning they are tailored to that layer’s size and sensitivity.

**Aggregation**: Upon receiving the encrypted local models from the participating clients, the central server proceeds with homomorphic aggregation on a per-layer basis. For each layer, the server collects the ciphertexts with layer-specific CKKS context. Using the additive homomorphism of CKKS, the server computes the sum of these ciphertexts without decrypting them. The aggregation is entirely performed in the encrypted domain. Thus, privacy is preserved by design, since no intermediate results are exposed in plaintext. This aligns with the goal of secure aggregation in FL; the server obtains only an encrypted combined update and cannot inspect the contribution of any single client. HEAT-FL’s server is context-aware in handling these operations. It maintains knowledge of each layer’s CKKS context and uses the appropriate values for that layer’s computations. In practice, this means the server runs separate homomorphic addition routines, each with the respective CKKS parameters.

**Model Update**: The server aggregates the securely updated parameters ($M_{L}^{*}$) from all clients to generate a new averaged encrypted global model ($M_{AG}^{*}$). This updated global model is then distributed back to all clients for the next round of training, as in PPFLMP implementation.

**Decryption**: In the decryption phase of the HEAT-FL framework, each client begins by receiving the encrypted global model $M_{AG}^{*}$ from the server. Since different layers of the model are encrypted using distinct CKKS contexts tailored to their size and sensitivity, the client must first identify the specific encryption context associated with each layer. Using its private key $s_{k}^{\prime }$, the client then decrypts each layer of $M_{AG}^{*}$. This ensures that the decryption process accurately reconstructs the plaintext values of the global model parameters. Once all layers are successfully decrypted, the client updates its local model $M_{L}$ accordingly, setting it as the initial state for the upcoming local training phase. This context-aware decryption mechanism allows the client to securely and efficiently retrieve the global model while preserving the privacy guarantees enforced by the adaptive encryption strategy.

Algorithm 2 presents the proposed HEAT-FL framework, which outlines the client and server-side operations for secure model aggregation.
**Algorithm 2:** HEAT-FL
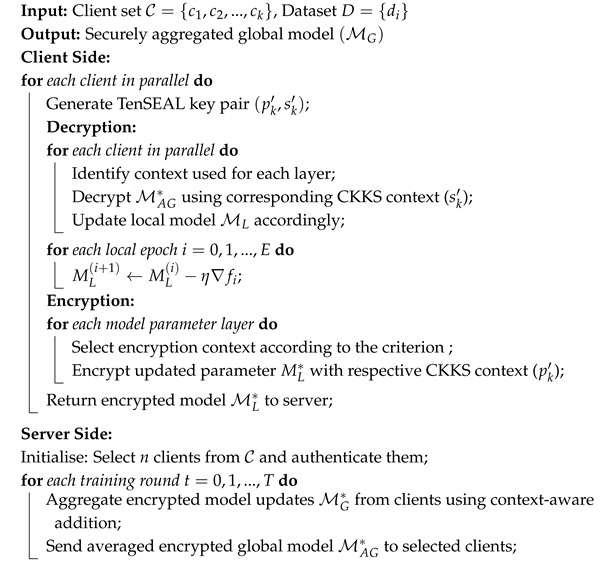


### 4.2. Baseline Comparisons

For a comprehensive evaluation, proposed HEAT-FL is compared with several established baseline techniques. These baselines are chosen to highlight and methodically improve the impact of federation and encryption on both model performance and system overhead:

1. Standard Federated Learning (FedAvg): This baseline is the vanilla federated learning approach using the FedAvg algorithm [26]. In standard FedAvg, multiple clients train locally on their data and periodically send model updates to a central server, which averages these updates to produce a new global model. No encryption or additional privacy mechanism is applied in this setup. This baseline isolates the effects of federated data distribution on model performance without any cryptographic overhead.
2. Paillier-based Federated Learning: For a baseline with privacy, we implemented a federated learning scheme using Paillier homomorphic encryption for secure aggregation following the methodology of prior research works [7].
3. CKKS/BFV-based Federated Learning: The PPFLMP framework adopts two lattice-based homomorphic encryption schemes, CKKS and BFV, to perform secure aggregation of encrypted model updates. Using both schemes under consistent experimental settings allows for a systematic evaluation of how encryption type influences model accuracy, computational load, and communication cost. This comparison offers practical insights into the privacy and performance trade-offs, supporting informed decision-making when selecting encryption methods for privacy-preserving federated learning systems.

These comparisons evaluate the privacy-performance trade-off of different homomorphic encryption schemes in federated learning contexts.

### 4.3. Datasets

To evaluate the effectiveness of the PPFLMP and HEAT-FL frameworks, we conducted experiments using Google Colab notebooks. In this study, we used PyTorch version 2.5.1+cu121 along with Torchvision version 0.20.1+cu121 for implementing and training deep learning models. This study utilizes two medical imaging datasets: the MRI Brain Tumor Dataset [27], containing MRI scans classified into normal, glioma, meningioma, and pituitary tumor classes, and the Chest X-ray Dataset [28], consisting of radiographs labelled as normal and pneumonia positive as depicted in Table 4. The primary focus of the experiments was to improve model accuracy with Federated Learning through secure model parameter aggregation and normalization while evaluating the respective communication overhead and computational efficiency.

We evaluated our framework using two distinct datasets to demonstrate performance across different use cases. The images are resized to 224 × 224 pixels, normalized to a [0, 1] range. Following preprocessing, all datasets are encrypted using HE encryption schemes before inference. The dataset is divided into 80% training and 20% testing, to ensure robust evaluation.

### 4.4. Model Architectures

The experiments were conducted in a homomorphic encryption-based federated learning environment with varying numbers of clients (2, 4, 6, 8, 10, 20) [29]. Training configuration included each client independently training a local model and encrypting its model updates before securely transmitting them to a central server for aggregation. Transfer learning was applied using a range of convolutional neural network models, including MobileNetV2, MobileNetV3, ResNet34, ResNet50, and DenseNet121 [30]. TenSeal’s CKKS homomorphic encryption was used to enable encrypted computations on model parameters, ensuring privacy preservation while facilitating secure arithmetic operations on ciphertexts. This approach allowed the server to perform aggregation without decrypting the individual model updates, resulting in normalized aggregated parameters that maintain both accuracy and security.

The secure aggregation implementation utilized TenSEAL’s serialization capabilities to minimize communication overhead, with model updates transmitted as encrypted byte arrays.

### 4.5. Evaluation Metrics

1. **Accuracy**: This measures the proportion of correct predictions made by the model. It is calculated as:
  (9) $$Accuracy=\frac{TP+TN}{TP+TN+FP+FN}$$
  where TP, TN, FP, and FN represent true positives, true negatives, false positives, and false negatives, respectively. Accuracy provides an overall indicator of model performance on the test data.
2. **Computational Overhead**: As edge devices in the healthcare domain are typically resource-constrained, computational overhead becomes an important metric in evaluating the feasibility of privacy-preserving federated learning frameworks. The overhead associated with each training round is systematically assessed by measuring the following key components:
  - Encryption time $T_{enc}$: The time it takes for each client device to encrypt its local model updates before sending them to the cloud. This reflects the client-side latency added by the homomorphic encryption process.
  - Decryption time $T_{dec}$: The time required to decrypt the aggregated model parameters. The decryption is performed collaboratively by the clients. In our implementation, this is the overhead for recovering the global model from its encrypted form at the end of each round.
  - Aggregation time $T_{agg}$: The time the server spends on aggregating the model updates while they are still encrypted. This involves homomorphic computations like adding and averaging encrypted parameters and reflects the server-side processing cost due to encryption.
3. **Communication Cost**: This metric captures the network and bandwidth overhead introduced by transmitting encrypted data. Homomorphic encryption increases the size of model updates due to the complexities of ciphertexts. It is important to understand how homomorphic encryption affects data communication [31].
  (10) $$C_{comm}=\sum_{k=1}^{K}\sum_{t=1}^{T}||E\left(w_{k}^{t}\right)\left|\right|$$
  where $\left||E(w_{k}^{t})|\right|$ represents the size of encrypted model parameters in bytes.

## 5. Results

This section presents a detailed analysis of model accuracy, computational and communicational efficiency, and the trade-offs inherent in the proposed HEAT-FL and PPFLMP framework using CKKS, BFV for healthcare applications.

### 5.1. Model Accuracy

**Figure 4:**
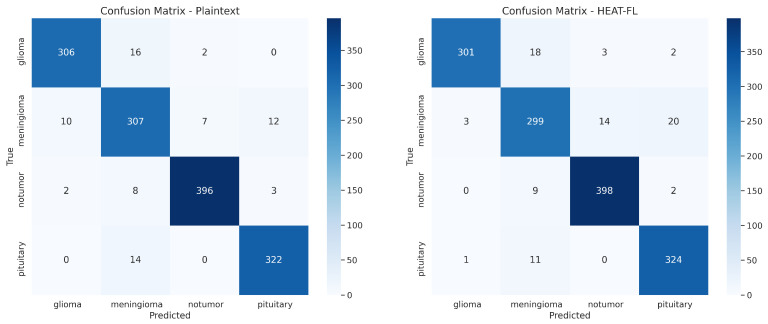
Confusion matrix-DenseNet121 model on brain tumor MRI images (Plaintext vs. HEAT-FL).

We systematically evaluated datasets [27,28] using MobileNetV2, MobileNetV3, ResNet34, ResNet50, and DenseNet121 models in a federated learning environment with CKKS homomorphic encryption scheme. This encryption process introduces noise into the numerical computations, which can affect model convergence patterns and final accuracy. However, our results demonstrate high classification accuracy while providing robust privacy guarantees. According to Table 5, within the encrypted adaptive federated learning setup, MobileNetV2 achieved 93.24% accuracy for pneumonia detection, while DenseNet121 reached 94.19% for brain tumor detection. Compared to plaintext federated learning baselines, the accuracy degradation introduced by the HEAT-FL framework remains within 0.5–1.5% across all model architectures, representing an excellent privacy-accuracy balance for sensitive healthcare applications. Figure 4 presents the confusion matrix for the DenseNet121 model applied to brain tumor MRI classification under both plaintext and HEAT-FL encrypted scenarios. Furthermore, a detailed evaluation using class-wise precision, recall, and F1-scores is provided in Table 6, highlighting the performance of the proposed HEAT-FL framework.

### 5.2. Computational Efficiency

**Figure 5:**
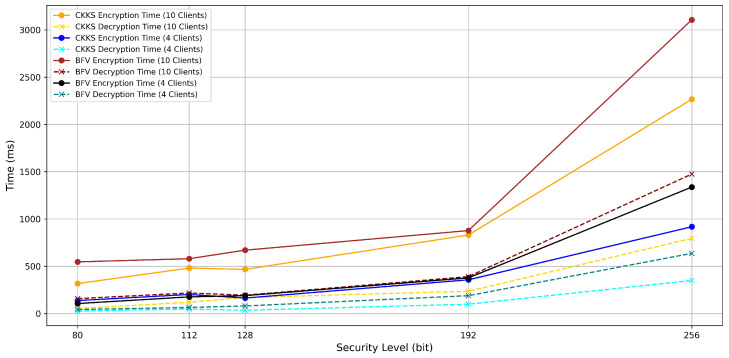
Encryption and decryption time per 1 federated round with respect to the bit security level (CKKS and BFV with PPFLMP).

**Figure 6:**
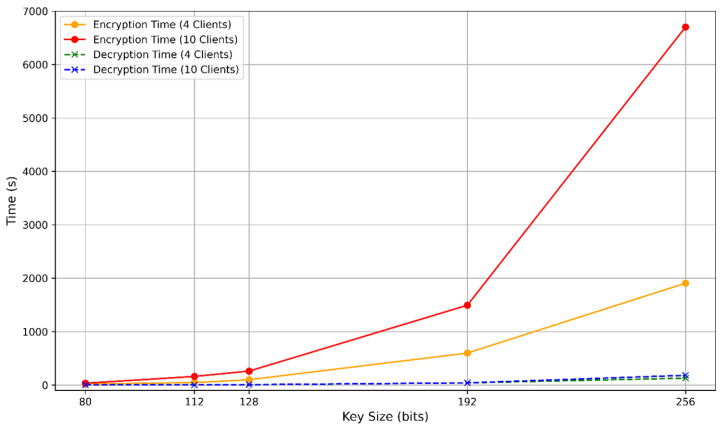
Encryption and decryption time per 1 federated round with respect to the bit security level (Paillier).

The computational overhead caused by homomorphic encryption (HE) in PPFLMP refers to the additional processing time required for encrypting, decrypting, and handling encrypted model parameters. To implement PPFLMP using TenSEAL version 0.3.16, the parameters were configured to match with standard security levels of 80, 112, 128, 192, and 256 bits. A custom lightweight model was used on the MRI Brain Tumor Dataset for the experiments due to time and computational resource constraints. As shown in Figure 5, encryption and decryption times increase with higher polynomial modulus sizes. This is expected, as greater security levels require larger key sizes, which result in more complex arithmetic operations on encrypted tensors. Since model parameters in federated learning are represented as tensors, the computational cost is dominated by both the dimensions of these tensors and the underlying HE scheme. Although encryption time increases with security level, the execution times remain within a feasible range measured in millisecond, making TenSEAL a practical choice for secure federated learning. For comparison, the Paillier encryption scheme was also tested at equivalent security levels using respective key sizes [12]. As illustrated in Figure 6, both encryption and decryption times increase substantially with key size, especially for higher security levels. Paillier exhibits a steeper rise in computational time compared to the CKKS and BFV schemes. This difference comes from the fundamental architectural design between the two schemes. TenSEAL, which is based on Learning With Errors (LWE), supports approximate computations over tensors and offers full homomorphic capabilities [25]. CKKS is capable of directly encoding floating-point tensors into ciphertext, while BFV requires integer conversions, which consume more computational costs. In contrast, Paillier is only partially homomorphic and lacks built-in support for tensor operations, requiring additional processing to adapt model parameters into compatible formats [32].

**Figure 7:**
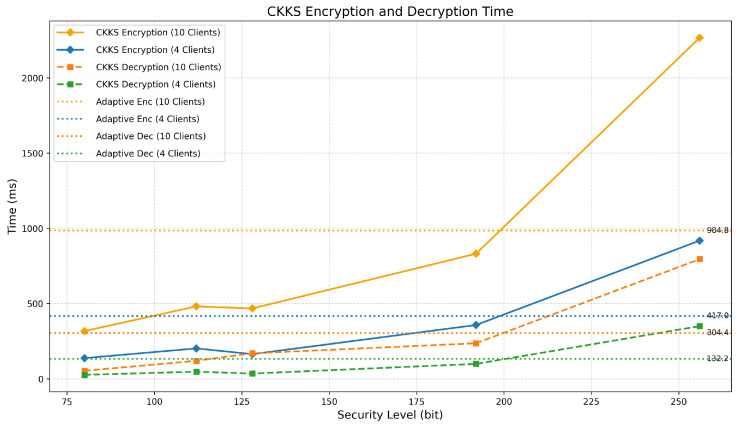
Encryption and decryption time per 1 federated round with respect to the bit security level (CKKS and HEAT-FL CKKS approach).

Building on the previous results, we evaluated the proposed adaptive CKKS versus plain CKKS encryption overhead. The adaptive approach assigns higher encryption priorities to final classification layers and initial feature extraction layers, as these layers are the most sensitive. Middle layers and activation function layers receive lower encryption priorities since they are considered less critical parameters. The comparison is shown in Figure 7. With this approach, we used the highest level of security for more critical layers of the model by reducing the overall computational burden. Compared to the fixed 256-bit security setting (polynomial modulus degree 32768), it achieves a 56.5% reduction in encryption time for 10 clients and 54.6% for 4 clients per one federated round.

**Figure 8:**
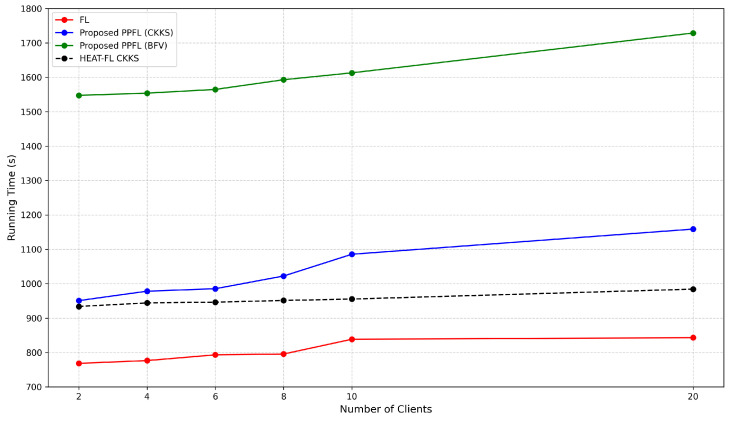
Running time to execute proposed frameworks with respect to the number of clients.

Figure 8 compares the total execution time of standard federated learning (FL) and PPFLMP using CKKS, BFV, and HEAT-FL CKKS, with respect to the number of participating clients under 256 bit-level security settings using the TenSEAL library. As shown, the PPFLMP-BFV framework consumes higher runtime than the framework based on CKKS, which is due to the additional operations performed based on integer transformation. However, the overhead generated by CKKS encryption schemes remains within acceptable limits and exhibits a linear growth pattern.

According to the above figure, the HEAT-FL CKKS approach reduces computational overhead compared to other approaches while maintaining the same high-level security configurations on critical model layers. Compared to the fixed 256-bit security setting in the PPFLMP (CKKS) method, the proposed HEAT-FL (Adaptive CKKS) approach achieves up to 12% reduction in total execution time for 10 clients.

### 5.3. Communicational Overhead

The communication cost increases with the implementation of PPFLMP and HEAT-FL frameworks, rather than a generic federated learning scenario, as the central server and the clients exchange the encrypted model parameters and aggregated model parameters in both directions. Table 7 presents the cost with respect to three security levels, considering two scenarios involving four and ten clients in a privacy-preserving federated learning setup. The mean communication cost per client increases with respect to the bit security level. This is because higher Polynomial Modulus Degree and Coefficient Modulus Bit Sizes result in higher ciphertext sizes. While these enhancements strengthen the security and allow precise computations, they also lead to a higher volume of data being transmitted between clients and the server.

## 6. Discussion

HEAT-FL reduces cryptographic overhead while preserving accuracy within 0.5–1.5% across all CNNs reported. Adaptive layer encryption framework reduces the encryption time by 56.5% with 10 clients and 54.6% with 4 clients per epoch relative to fixed parameter CKKS. In practice, these performance attributes translate to faster and secure learning on image data analysis in hospital networks. In parallel, the adaptive framework minimizes unnecessary ciphertext growth on low-sensitivity layers.

Despite these improvements, HEAT-FL still incurs compute and communication costs that scale with the number of clients, model depth, and the target adaptive security levels. Ciphertext size can become a bottleneck for deeper model layers with a large number of clients. The selected encryption parameters were optimized for image classification in the context of pneumonia detection from X-ray images and brain tumor detection from MRI scans. A potential extension of this work involves conducting a detailed analysis of how different sensitivity thresholds for model layers influence the trade-off between privacy and efficiency.

## 7. Conclusions

The proposed HEAT-FL framework emerges as a promising approach for balancing security in sensitive layers with optimized computational and communicational overhead through context-aware parameter tuning over other PPFLMP frameworks. According to the results, this approach outperforms the computational and communication overhead of fixed bit security settings while maintaining the same level of security for the most sensitive and important model layers. Within the PPFLMP framework, CKKS homomorphic encryption demonstrates superior performance for tensor-based model parameters, delivering faster runtime and enhanced scalability compared to BFV and Paillier schemes. Despite the security advancements of these cryptographic frameworks, their clinical deployment may face several practical and regulatory barriers. One significant challenge is the lack of validation and regulatory approval for advanced cryptographic methods and the use of federated learning, which are not yet clearly addressed in most healthcare compliance frameworks. Future work will also focus on developing a Hybrid Homomorphic Encryption (HHE) model suitable for cloud-edge integration by combining symmetric encryption techniques such as PASTA with homomorphic encryption, aiming to achieve an optimal balance between robust security and reduced computational and communication costs.

## Figures and Tables

**Table 1:** Notation Summary for Homomorphic Encryption and Federated Learning.

Symbol	Description
E(·)	Encryption function
D(·)	Decryption function
⊕	Homomorphic addition operation
⊗	Homomorphic multiplication operation
Δ	Scaling factor used in CKKS for precision control
$F_{k}\left(w\right)$	Local objective function of client *k*
F(w)	Global objective function aggregated across all clients

**Table 2:** Comparison of cryptographic schemes used in the research [17].

Feature	Paillier	BFV	CKKS
Tensor compatibility	Integers (scaled floats)	Integers (scaled floats)	Floating-point
Precision	Exact	Exact	Approximate
Multiplication support	Supports ciphertext- constant multiplication	Full	Full
Quantum resistance	No	Yes	Yes
Hardness Assumption	Decisional Composite Residuosity (DCRA), Integer factoring	Ring-Learning with Errors (RLWE), Lattice Problem	Ring-Learning with Errors (RLWE), Lattice Problem
Client overhead	High-small scale operations, Low-batched tensor operations	Moderate	Moderate
Use cases	Lightweight operations ex-voting, aggregation	Exact updates	Deep learning

**Table 3:** Configuration Details for Polynomial Modulus and Scale. (This table summarizes the parameter configurations for CKKS encryption, including polynomial modulus degrees, coefficient modulus bit sizes, and scale values. The settings are selected to meet varying post-quantum security levels (80-bit to 256-bit) as recommended by NIST Security Guidelines and TenSEAL Documentation [24,25]).

Configuration	Polynomial Modulus Degree	Coefficient Modulus	Scale
Set 1 (80-bit security level)	8192	[50, 40, 50]	$2^{40}$
Set 2 (112-bit security level)	8192	[40, 40, 40, 40, 40]	$2^{40}$
Set 3 (128-bit security level)	8192	[60, 40, 40, 40, 60]	$2^{40}$
Set 4 (192-bit security level)	16,384	[60, 40, 40, 40, 40, 60]	$2^{40}$
Set 5 (256-bit security level)	32,768	[60, 40, 40, 40, 40, 40, 40, 60]	$2^{40}$

**Table 4:** Dataset Overview.

Dataset	Total Samples	Classes
Chest X-ray Dataset [28]	5840 (5216 Train, 624 Test)	2 (Normal, Pneumonia)
MRI Brain Tumor Dataset [27]	7022 (Train: 5711, Test: 1311)	4 (Glioma, Meningioma, Normal, Pituitary)

**Table 5:** Classification accuracy Comparison (Plaintext vs. HEAT-FL).

Model	Pneumonia Detection (%)		Brain Tumor Detection (%)	
	Plaintext	HEAT-FL	Plaintext	HEAT-FL
MobileNetV2	94.41	93.23	91.33	90.18
MobileNetV3	88.43	87.21	83.79	82.85
ResNet34	93.65	92.47	91.78	90.82
ResNet50	93.15	91.91	90.94	90.11
DenseNet121	91.73	90.74	94.60	94.09

**Table 6:** Class-wise Precision, Recall, and F1-Score for Pneumonia and Brain Tumor Detection using HEAT-FL.

Model	Class	Precision	Recall	F1-Score	Class	Precision	Recall	F1-Score
MobileNetV2	Covid	0.9578	0.9020	0.9289	Glioma	0.9962	0.8120	0.8943
	Normal	0.9119	0.9623	0.9364	Meningioma	0.7635	0.8839	0.8192
					NoTumor	0.9603	0.9462	0.9532
					Pituitary	0.9169	0.9524	0.9343
MobileNetV3	Covid	0.9592	0.7699	0.8543	Glioma	0.8077	0.8426	0.8248
	Normal	0.8167	0.9691	0.8864	Meningioma	0.7390	0.5923	0.6572
					NoTumor	0.8348	0.9585	0.8927
					Pituitary	0.9175	0.8588	0.8876
ResNet34	Covid	0.9015	0.9489	0.9246	Glioma	0.9587	0.8673	0.9103
	Normal	0.9490	0.9016	0.9247	Meningioma	0.8147	0.8235	0.8191
					NoTumor	0.9464	0.9682	0.9572
					Pituitary	0.9090	0.9583	0.9330
ResNet50	Covid	0.9039	0.9333	0.9183	Glioma	0.9727	0.8241	0.8924
	Normal	0.9347	0.9065	0.9203	Meningioma	0.8741	0.9280	0.9003
					NoTumor	0.9346	0.9789	0.9558
					Pituitary	0.9006	0.9702	0.9339
DenseNet121	Covid	0.8519	0.9801	0.9119	Glioma	0.9873	0.9284	0.9569
	Normal	0.9780	0.8383	0.9024	Meningioma	0.8878	0.8907	0.8892
					NoTumor	0.9588	0.9787	0.9686
					Pituitary	0.9310	0.9643	0.9469

**Table 7:** Communication Overhead Comparison of CKKS and HEAT-FL CKKS for Different Numbers of Federated Clients ($N_{c}$).

$N_{c}$	128-bit		192-bit		256-bit	
	CKKS	HEAT-FL CKKS	CKKS	HEAT-FL CKKS	CKKS	HEAT-FL CKKS
4	13,372.49 kB	10,430.54 kB	33,714.91 kB	25,623.33 kB	92,765.04 kB	73,284.38 kB
10	33,429.50 kB	26,076.35 kB	84,288.03 kB	64,058.32 kB	231,928.73 kB	183,210.95 kB

## Data Availability

The original data presented in the study are openly available in Kaggle at https://www.kaggle.com/datasets/paultimothymooney/chest-xray-pneumonia and https://www.kaggle.com/datasets/masoudnickparvar/brain-tumor-mri-dataset.

## Abbreviations

The following abbreviations are used in this manuscript:

MLaaS	Machine Learning as a Service
HIPAA	Health Insurance Portability and Accountability Act
GDPR	General Data Protection Regulation
FL	Federated Learning
HE	Homomorphic Encryption
SEAL	Simple Encrypted Arithmetic Library
CKKS	Cheon–Kim–Kim–Song
BFV	Brakerski–Fan–Vercauteren
PPFLMP	Privacy-Preserving Federated Learning Model Parameter framework
HEAT-FL	Homomorphic Encryption-based Adaptive Tuning for Federated Learning framework
